# Detecting liver fibrosis with Gd-EOB-DTPA-enhanced MRI: A confirmatory study

**DOI:** 10.1038/s41598-018-24316-z

**Published:** 2018-04-18

**Authors:** Niklas Verloh, Kirsten Utpatel, Michael Haimerl, Florian Zeman, Lukas Beyer, Claudia Fellner, Frank Brennfleck, Marc H Dahlke, Christian Stroszczynski, Matthias Evert, Philipp Wiggermann

**Affiliations:** 1 Department of Radiology, University Hospital Regensburg, Regensburg, Germany; 2 Department of Pathology, University Regensburg, Regensburg, Germany; 3 Centre for Clinical Trials, University Hospital Regensburg, Regensburg, Germany; 4 Department of Surgery, University Hospital Regensburg, Regensburg, Germany

## Abstract

Strong correlations between the grade of fibrosis and cirrhosis, classified using the Ishak scoring system, and the uptake characteristics of Gd-EOB-DTPA with the relative enhancement (RE) of the liver parenchyma have been reported. To confirm the results of a retrospective analysis, patients undergoing liver surgery were prospectively examined with Gd-EOB-DTPA-enhanced liver 3 Tesla MRI to determine the degree of liver fibrosis. Correlations between the grade of fibrosis and cirrhosis, classified using the Ishak scoring system, and RE were investigated and compared with those derived from an initial retrospective study. After validating the cut-off values in the retrospective study (Ishak ≥ 1, RE-cut-off 0.90; Ishak ≥ 2, RE-cut-off 0.79; Ishak ≥ 4, RE-cut-off 0.60; and Ishak = 6, RE-cut-off 0.47), we showed that Gd-EOB-DTPA has a high sensitivity (≥86%) and a high positive predictive value (≥86%). These results support the use of Gd-EOB-DTPA-enhanced liver MRI as a non-invasive method for determining the degree of liver fibrosis and cirrhosis.

## Introduction

Chronic liver disease and cirrhosis are leading causes of mortality in the Western hemisphere. The epidemic increase in obesity, nonalcoholic fatty liver disease and alcohol-induced liver cirrhosis contribute to this growing problem, as morbidity and mortality are directly correlated with the progression of hepatic fibrosis^1–5^.

Information regarding the grade of liver fibrosis and cirrhosis is essential for determining the prognosis and clinical management of patients with a chronic liver disease or patients who undergo liver surgery^6,7^.

Liver fibrosis and cirrhosis are currently considered to be dynamic processes that can be corrected with adequate treatment^8^. In clinical practice, obtaining a liver biopsy is the gold standard for monitoring the state of liver fibrosis and observing treatment response.

However, liver biopsies are an invasive procedure known to have poor patient compliance. Biopsies are also prone to both misinterpretation in cases of missing fibrotic septa or nodular configurations and to inter-observer variability^9,10^. The quality of the grading is directly correlated with the sampling size. Furthermore, liver fibrosis may cause heterogeneity of the liver tissue; therefore, sampling results or faulty sampling in liver biopsy may not be representative of the whole organ^10–12^. An image-based technique would be helpful, as it would allow not only a small section of the liver but also the entire organ to be examined.

Upper abdominal ultrasonography is a common tool for analysing liver stiffness with elastography^13^. However, ultrasound examinations are examiner-dependent and limited by a restricted field of view, and they are therefore not usually able to assess the entire liver.

Non-invasive assessment of liver fibrosis is an important area of study. Multiple techniques have been proposed, such as elastography. Specifically, area under the curve (AUC) analyses of MR elastography showed a diagnostic accuracy of 90.9–99.4%. The corresponding values for US-based vibration-controlled transient elastography have been reported to be 83.7–91.4%^14–16^. One disadvantage of these examinations is that liver stiffness is only an indirect sign of liver fibrosis; concomitant diseases, such as heart failure, also affect liver stiffness. Thus, elastography techniques are prone to mismeasurements. In addition to those limitations, some of the abovementioned examinations require additional equipment, limiting the applicability and the routine use of these techniques in clinical practice.

MRI examinations with hepatocyte-specific MR contrast agents are used in routine clinical practice for hepatic lesion detection and differentiation. In addition to the general assessment of tissue perfusion in the vascular phases, these contrast agents allow an assessment of the liver parenchyma during an additional late phase (hepatobiliary phase, HBP) due to their specific accumulation in hepatocytes. As the hepatic accumulation is dependent on the integrity of the hepatocytes, quantification of the uptake with mean values of relative enhancement (RE) can be used to analyse the liver parenchyma^17–23^.

Studies have shown that RE, representing the hepatic uptake of Gd-EOB-DTPA, is strongly affected by the degree of liver cirrhosis, represented by clinical scoring systems such as the Child Pugh Score^17,24^. The enhancement patterns of the liver parenchyma in Gd-EOB-DTPA-enhanced MRI even correlate with the graft survival probability in liver transplant recipients^25^.

Recently published studies have shown a wide range in diagnostic accuracy regarding the ability of analysing the underling histopathological data of the liver parenchyma in retrospective studies with different parenchymal enhancement measurements^26–29^. These studies agree that the uptake of Gd-EOB-DTPA and, thus, the relative enhancement of HBP compared with baseline may be delayed in the case of liver fibrosis^27,28^. In contrast, these results can be used to determine the degree of liver fibrosis and cirrhosis. Feier *et al*. reported an association between RE, representing the hepatic uptake of Gd-EOB-DTPA, and the METAVIR scoring system for differentiating normal liver parenchyma and higher stages of liver fibrosis^26^.

Recently, we published a retrospective study correlating liver fibrosis with histopathological findings, analysed using the Ishak scoring system. In this study, we were able to show that a strong correlation exists between the uptake of Gd-EOB-DTPA and fibrosis severity. With a diagnostic accuracy of 93–98%, we showed that liver fibrosis can be demonstrated by the RE^29^.

The aim of this study was to validate the retrospective finding by a confirmatory study in patients undergoing liver resection and transplantation.

## Results

### Comparison of the different stages of liver fibrosis

A comparison of the patients with no liver fibrosis (NLF) and those with liver fibrosis (LF) stratified according to the Ishak classification revealed that RE decreased together with the extent of liver fibrosis.

**Figure 1:**
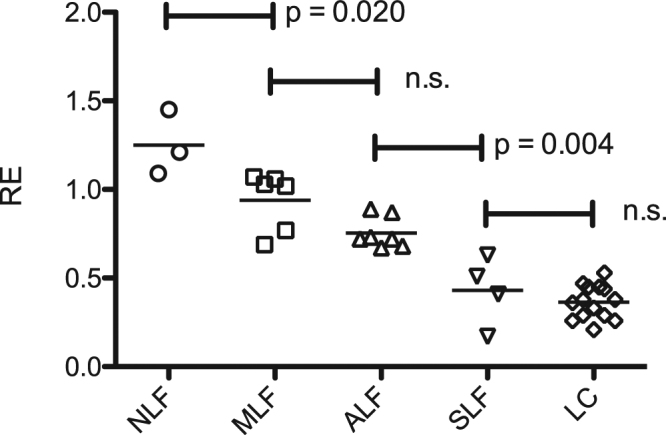
Scatter plot of the different stages of liver fibrosis. The relative enhancement (RE) according to the Ishak scores with the corresponding p values. NLF, no liver fibrosis; MLF, mild liver fibrosis; ALF, advanced liver fibrosis; SLF, severe liver fibrosis; LC, liver cirrhosis.

In adjusted pairwise comparisons (Fig. 1, Table 1), significant differences were observed between patients without fibrosis (NLF, Ishak = 0; RE, 1.25 ± 0.18) and mild liver fibrosis (MLF, Ishak = 1; RE, 0.94 ± 0.17) (p = 0.020), as well as between patients with ALF and severe liver fibrosis (SLF, Ishak 4 + 5; RE, 0.43 ± 0.20) (p = 0.004). However, no significant differences were observed between patients with MLF and advanced liver fibrosis (ALF, Ishak 2 + 3; RE, 0.75 ± 0.08) (p = 0.150) and between patients with SLF and liver cirrhosis (LC, Ishak = 6 RE, 0.36 ± 0.10) (p = 1.000).

**Table 1:** Differentiation between the stages of liver fibrosis.

	NLF	MLF	ALF	SLF	LC
NLF		0.020	≤0.001	≤0.001	≤0.001
MLF	0.020		0.150	≤0.001	≤0.001
ALF	≤0.001	0.150		0.004	≤0.001
SLF	≤0.001	≤0.001	0.004		1.000
LC	≤0.001	≤0.001	≤0.001	1.000	

Comparison of the different stages of liver fibrosis with the corresponding p values. NLF, no liver fibrosis; MLF, mild liver fibrosis; ALF, advanced liver fibrosis; SLF, severe liver fibrosis; LC, liver cirrhosis.

### Comparison of the confirmatory study and the initial retrospective findings

**Figure 2:**
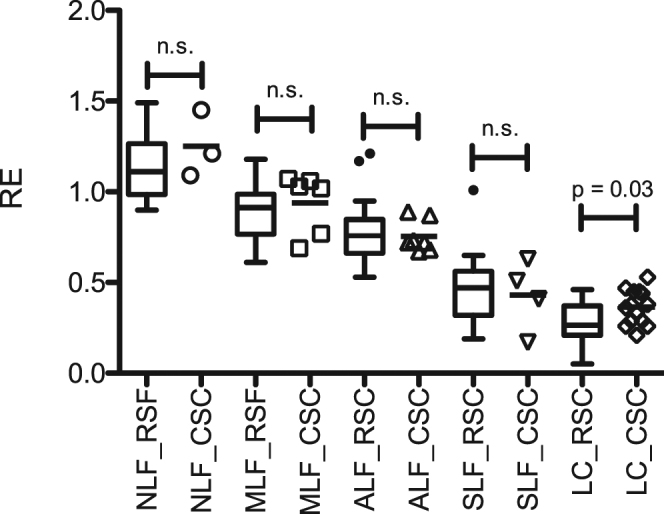
Comparison of the retrospective and prospective findings. Boxplots of the initial retrospective (RSF) study compared with the results of the prospective, confirmatory study cohort (CSC). The relative enhancement (RE) for every patient in the CSC is shown together with the corresponding statistical significance of differences between the two study groups for the different stages of liver fibrosis. NLF, no liver fibrosis; MLF, mild liver fibrosis; ALF, advanced liver fibrosis; SLF, severe liver fibrosis; LC, liver cirrhosis.

In a comparison with the initial results, we found no significant difference between patients with NLF, MLF, ALF and SLF (Fig. 2). Only patients with LC showed a higher RE in the current analysis than in the previously published findings.

### Validating the cut-off values

We initially described four cut-off values for analysing liver fibrosis: MLF or greater (Ishak ≥ 1, RE-cut-off 0.90), ALF or greater (Ishak ≥ 2, RE-cut-off 0.79), SLF or greater (Ishak ≥ 4, RE-cut-off 0.60), and LC (Ishak = 6, RE-cut-off 0.47).

**Figure 3:**
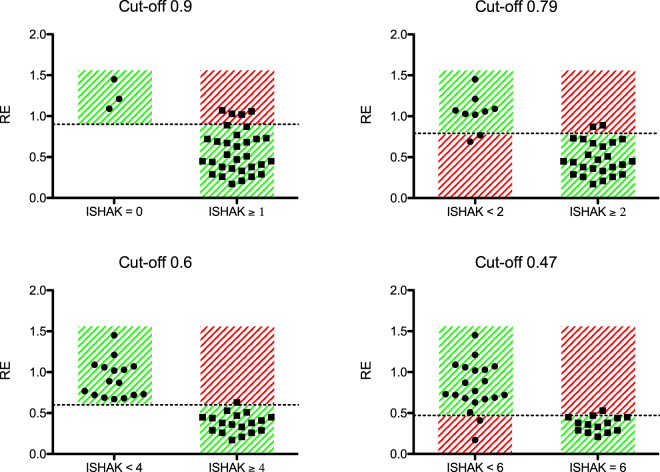
Cut-off values. The cut-off values of the retrospective study were applied to the prospective study cohort. The graphs show the findings for the prospective study cohort. The dashed line indicates the cut-off value of the relative enhancement (RE). The green area shows the correctly assigned patients, and the red area shows the negatively assigned patients.

The cut-off values were applied to the prospective data. Table 2 shows the resulting sensitivities and specificities and the corresponding positive and negative predictive values. The sensitivity for detection of initial stage of liver fibrosis compared to no liver fibrosis (Ishak ≥ 1) was 0.87; the corresponding specificity was 1.00. Differentiation of full liver cirrhosis (Ishak = 6) compared to other stages of liver fibrosis was 0.86, with a specificity of 0.90. Figure 3 is a graphical representation of the data.

**Table 2:** Validating the cut-off values.

	Sensitivity	Specificity	Positive predictive value	Negative predictive value
Ishak ≥ 1	0.87	1.00	1.00	0.42
Ishak ≥ 2	0.92	0.78	0.92	0.78
Ishak ≥ 4	0.94	1.00	1.00	0.94
Ishak = 6	0.86	0.90	0.86	0.90

The sensitivity and specificity and the corresponding positive and negative predictive values are shown for the different cut-off values. MLF or greater (Ishak ≥ 1, RE-cut-off 0.90), ALF or greater (Ishak ≥ 2, RE-cut-off 0.79), SLF or greater (Ishak ≥ 4, RE-cut-off 0.60), and LC (Ishak = 6, RE-cut-off 0.47).

## Discussion

The non-invasive assessment of liver cirrhosis is of high clinical relevance. In this prospective trial, the use of Gd-EOB-DTBA-enhanced MRI to assess the degree of liver fibrosis, expressed as the histological Ishak score, was examined to validate the findings of our initial retrospective study.

Patients showed a continuous decrease in RE with increasing Ishak score and significant differences between NLF and ALF in a paired comparison.

Excluding patients with liver cirrhosis, we were unable to find a significant difference between the prospective and retrospective patient groups. Patients with liver cirrhosis showed a significantly higher RE than the initial retrospective group. This finding may be due to the similarity of the histopathological findings in patients with SLF and LC. Chen *et al*. recently showed the divergence in inter-observer agreement (ICC 0.89) between two histopathological interpretations, with a difference of a least one fibrosis stage in 30% of the cases^30^.

Validating the cut-off values of the retrospective study showed that Gd-EOB-DTPA has a high sensitivity (≥86%) with a high positive predictive value (≥86%) for any stage of liver fibrosis.

For the initial liver fibrosis, contrast-enhanced-MRI with a cut-off of RE = 0.9 showed a high positive predictive value of 100%; however, the negative predictive value was poor at only 42% (sensitivity 87%, specificity 100%), which may be accounted for by the small number of patients with no liver fibrosis included in the study. The data demonstrate that contrast-enhanced MRI, with a cut-off value of RE = 0.6, is very suitable for staging a high degree of liver fibrosis (Ishak ≥ 4), with a positive predictive value of 100% and a negative predictive value of ≥94% (sensitivity 94%, specificity 100%).

The limiting factors of our study are the single centre design, a small number of patients and especially the limited number of patients in each non-cirrhotic class. Nevertheless, we were able to show that MRI potentially is a sensitive, non-invasive, method for determining the degree of liver fibrosis. Furthermore, as an image-based modality evaluating the entire liver, MRI offers the advantage of reducing the risk of misinterpretation or sampling errors.

Further multicentre studies are needed to confirm the results of this single-centre study. The addition of new MRI methods, like texture analysis, might further enhance the presented results.

## Methods

We followed the study design of Verloh *et al*.^29^.

### Patients

Approval from the local institutional review board of the University Hospital Regensburg was obtained for this prospective study. This study was registered retrospectively with the German Clinical Trails Register (DRKS00012564, Date of Registration: July 18, 2017) and performed in accordance with the relevant guidelines and regulations. Written informed consent was obtained from the study participants.

**Figure 4:**
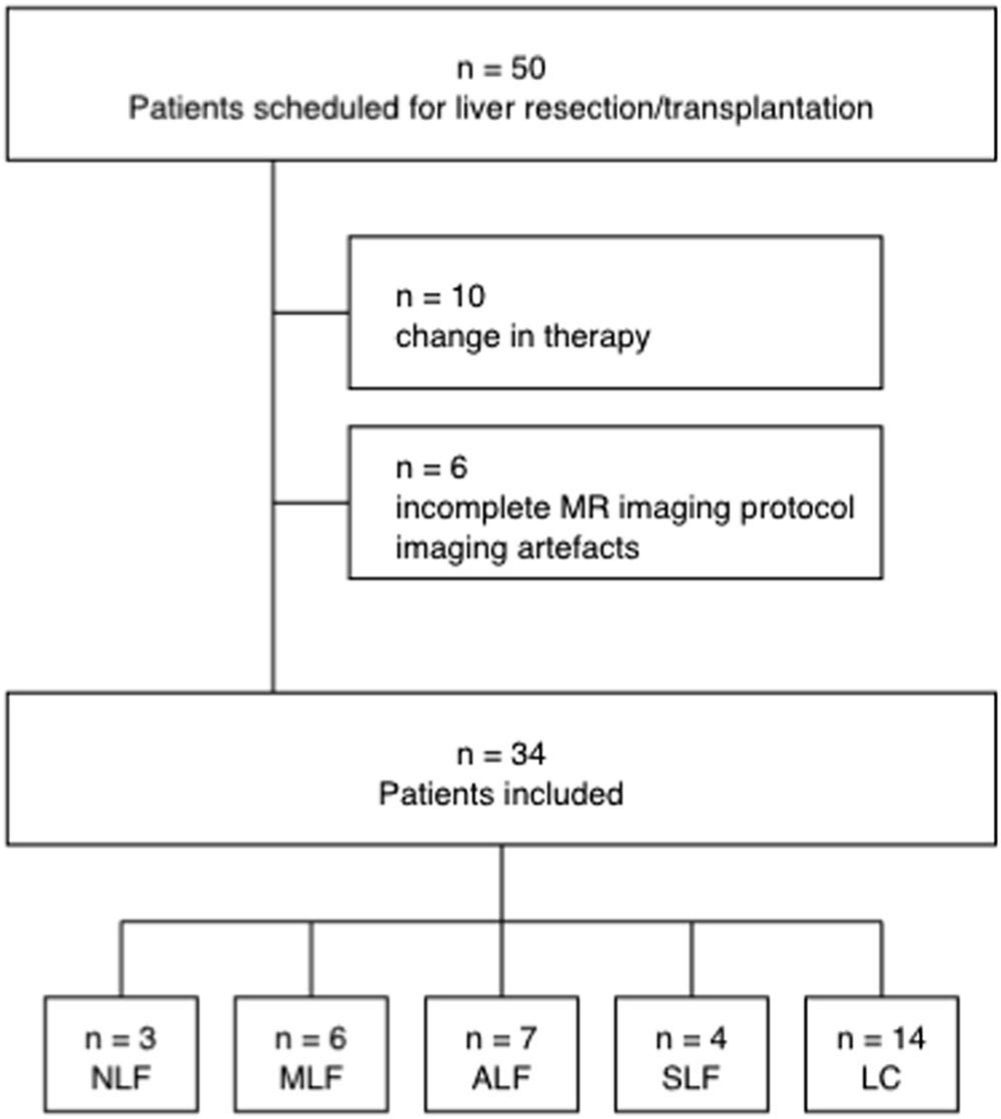
Flowchart of the inclusion and exclusion of study patients. The patients were included or excluded and allocated to different categories according to the Ishak score. NLF, no liver fibrosis; MLF, mild liver fibrosis; ALF, advanced liver fibrosis; SLF, severe liver fibrosis; LC, liver cirrhosis.

Overall, 50 adult patients were included in this study between August 2016 and April 2017. Ten patients were excluded from this study due to changes in therapy, e.g., no liver resection or atypical liver resection with an insufficient liver sample. Additionally, 6 patients were excluded due to the inability to complete the full MRI protocol and the presence of severe imaging artefacts due to surgical clips or poor breath-holding techniques. Finally, 34 patients (23 men and 11 women; mean age, 60 ± 11 years) were included in our analysis (Fig. 4).

All patients underwent Gd-EOB-DTPA-enhanced MRI of the liver before liver resection and transplantation. The patients underwent liver surgery for liver transplantation or for resection in cases of metastasis or liver cancer.

None of the recruited patients had any contraindications for MRI examination (e.g., claustrophobia, incompatible metallic implants, and pacemakers), contraindications for the administration of Gd-EOB-DTPA (e.g., renal failure as defined by a glomerular filtration rate < 30 ml/min) or a previous reaction to liver-specific MRI contrast agents.

### MRI

All imaging was performed using a clinical whole-body 3-T system (Magnetom Skyra, Siemens Healthcare) and combination body-spine array coil elements (18-channel body matrix coil and 32-channel spine matrix coil) for signal reception. Each of the T1-weighted volume-interpolated breath-hold examination (VIBE) sequences with fat suppression (repetition time (TR), 3.09 ms; echo time (TE), 1.16 ms; flip angle, 9°; parallel imaging factor, 2; slices, 64; reconstructed voxel size, 1.3 × 1.3 × 3.0 mm; measured voxel size, 1.7 × 1.3 × 4.5 mm; acquisition time, 14 s) covered the entire liver, and the sequences were applied before (non-contrast) and 20 min after contrast injection (HBP). Each sequence was acquired during one breath hold, and no additional system adjustments were performed for the post-contrast sequence.

All patients received a body weight-adapted dose (0.025 mmol/kg body weight) of Gd-EOB-DTPA (Primovist, Eovist; Bayer Schering Pharma AG, Berlin, Germany). The hepatocyte contrast agent Gd-EOB-DTPA was administered via a bolus injection at a flow rate of 1 ml/s and flushed with 20 ml of NaCl solution.

### Image analysis

Three regions of interest (ROIs) were manually selected in each liver lobe (with identical sizes and locations in the non-contrast and post-contrast T1 HBP) by an experienced physician (5 years of experience in hepatobiliary imaging). ROIs were manually adjusted to adapt to different respiratory levels or patient movement. Visible vessels, imaging artefacts and liver lesions were excluded. The sizes of the ROIs ranged from 1.0 to 2.5 cm^2^.

The mean SI was calculated and used as the representative SI for the entire liver. The RE between non-contrasted (SI_pre_) and post-Gd-EOB-DTPA (SI_post_) was calculated as follows:

1 $$\,{\rm{Relative}}\,{\rm{enhancement}}\,({\rm{RE}})\,of\,signal\,intensity\,(SI)=\frac{S{I}_{post}-S{I}_{pre}}{S{I}_{pre}}$$

The RE was used to determine the uptake of Gd-EOB-DTPA into the liver parenchyma and was correlated with the histopathological findings. The image was analysed blindly without knowledge of the histopathological examination.

### Histopathological examination

In the present study, only liver samples from liver resection (n = 30) or transplantation (n = 4) were included to avoid the limitation of an insufficient tissue sample, making the Ishak classification more reliable. Four-micrometre-thick sections were cut vertically and mounted on glass slides. Thereafter, the sections were deparaffinised with xylene and ethanol and stained with haematoxylin-eosin (HE) and Elastica van Gieson (EVG) according to standard protocols. EVG staining was used to evaluate LF. Collagen stained red, and the hepatocytes stained yellow.

Two pathologists (M.E. and K.U.) who specialize in liver histopathology reviewed the resection specimens to evaluate the degree of specific fibrosis and cirrhosis. Both examiners were blinded to the imaging results and the patient data. The scoring was performed independently. In cases of disagreement, additional microscopic analyses were performed in consensus. The fibroses were classified using the Ishak scoring system^31^. The patients were subdivided into the following 5 categories: NLF (Ishak 0; n = 3), MLF (Ishak 1; n = 6), ALF (Ishak 2 + 3; n = 7), SLF (Ishak 4 + 5; n = 4), and LC (Ishak 6; n = 14).

### Statistical analysis

All statistical analyses were performed with IBM SPSS Statistics (version 24, Chicago, IL, USA) and R 3.2.1^32^. The data are presented as the mean ± standard deviation (SD). We used the non-parametric Mann-Whitney U-test for independent variables for comparisons between the groups. To compare the relative enhancement between the Ishak stages, an analysis of variance (ANOVA) followed by Bonferroni adjusted pairwise comparisons was performed. REs at identical Ishak stages were compared in the retrospective and prospective studies with the non-parametric Mann-Whitney U-test.

Finally, the sensitivity, specificity, and positive and negative predictive values for classifying the Ishak score were calculated using the previously described four cut-off values for liver fibrosis analysis: MLF or greater (Ishak ≥ 1, RE-cut-off 0.90), ALF or greater (Ishak ≥ 2, RE-cut-off 0.79), SLF or greater (Ishak ≥ 4, RE-cut-off 0.60), and LC (Ishak = 6, RE-cut-off 0.47)^29^. These cut-off values were estimated according to the Youden indices in the initial retrospective data set based on ROC analyses of the patient groups.

All tests were two-sided, and p values < 0.05 indicated a statistically significant difference.

### Data availability

The data that support the findings of this study are available from the corresponding author upon reasonable request.

## Electronic supplementary material

Trial Description
