# Supplementary material for: Detecting liver fibrosis with Gd-EOB-DTPA-enhanced MRI: A confirmatory study
Source: Sci Rep. 2018 Apr 18;8:6207. doi: 10.1038/s41598-018-24316-z (PMC5906481; doi:10.1038/s41598-018-24316-z)
Supplement: Supplementary file 1 — Trial Description [file 41598_2018_24316_MOESM1_ESM.docx]

Prospective study on magnetic resonance tomography (MRI)-based molecular liver parenchyma diagnostics

Project summary

Liver cirrhosis is an advanced stage of liver fibrosis. Furthermore, it defined as a destruction of the lobules and vessel architecture and a nodular regeneration of the liver parenchyma. The process of liver fibrosis and cirrhosis is considered a dynamic process nowadays: liver cirrhosis can be recompensated under adequate therapy of the underlying disease.

Test that predicts liver function can lead to customized patient treatment. However, the liver performs several biochemical functions, making functional test hard to perform. Usually, only sections of the liver functions can be detected with biochemical test methods. Clinical test procedures have in common that only the global liver function can be measured. These methods fail when identifying regional dysfunctions when they are compensated. Image-based methods offer the advantage that they can represent not only the global but also the regional liver function.

The aim of this project is to evaluate prospectively the extent to which it is possible to determine the degree of liver fibrosis/cirrhosis and liver function with the aid of contrast-enhanced magnetic resonance tomography.

For this purpose, additional MRI sequences are performed in patients with planned elective liver resection, undergoing a scheduled MRI examination as part of the preoperative preparation.

The obtained data are to be correlated with the grade of the liver fibrosis/cirrhosis, classified using the Ishak scoring system.

General information

Organizational Data

- DRKS-ID:  DRKS00012564
- Date of Registration in DRKS:  2017/07/18
- Investigator Sponsored/Initiated Trial (IST/IIT):  yes

Secondary IDs

- Universal Trial Number (UTN):   U1111-1197-4450

Health Condition or Problem studied

- ICD10: K74 -  Fibrosis and cirrhosis of liver

Characteristics

- Study Type:  Interventional
- Allocation:  Single arm study
- Blinding:  Blinded
- Who is blinded:  assessor
- Control:  Uncontrolled/Single arm
- Purpose:  Diagnostic
- Assignment:  Single (group)
- Phase:  N/A
- Off-label Drug use:  N/A

Countries of Recruitment

- DE:   Germany

Locations of Recruitment

- University Hospital Regensburg

Addresses

- Primary Sponsor
  University Hospital Regensburg
  Franz-Josef-Strauß-Allee 11
  93053 Regensburg, Germany
- Contact for Scientific Queries
  University Hospital Regensburg
  Dr.  Niklas Verloh 
  Franz-Josef-Strauß-Allee 11
  93053 Regensburg, Germany
- Contact for Public Queries
  University Hospital Regensburg
  Ms.  Stefanie Hagen 
  Franz-Josef-Strauß-Allee 11
  93053 Regensburg, Germany

Sources of Monetary or Material Support

- Institutional budget, no external funding (budget of sponsor/PI)

Rationale & background information

Liver fibrosis and its progression to cirrhosis of the liver is of enormous importance in hepatobiliary medicine. Test that predicts liver function can lead to customized patient treatment to prevent the process of the fibrosis progression. There is currently no noninvasive test procedure, which can make a statement about the liver parenchyma and its function in the whole as well as in the regional.

The liver biopsy currently represents the gold standard in for the diagnosis and assessment of liver fibrosis/cirrhosis. Liver biopsy is an invasive procedure and has a poor patient satisfaction, is prone to random errors, is subject to interobserver variability, and offers the risk of complications such as infections or bleeding [1]. If characteristic fibrotic septa and nodular configurations are absent, the histological diagnosis of liver fibrosis, cirrhosis can be difficult [2]. Furthermore, the severity of the disease can be underestimated in the event of a faulty sample collection [3,4].

In the imaging of the liver, upper abdomen sonography is to be mentioned as an apparatus diagnostic in the determination of liver function. With the aid of elastography the liver stiffness can be measured, and thus a statement about the degree of fibrosis can be made indirectly [5,6]. However, due to false measurements as well as by the character of a dynamic investigation, certain limitations are obtained: Particularly to be mentioned here are the limited reproducibility and the dependence on the examiner [7].

In addition to imaging by means of ultrasound, nowadays the MRI of the liver with hepatocyte-specific MR contrast medium is to be mentioned. Gd-EOB-DTPA is a hepatocyte-specific paramagnetic MR contrast agent. It can be used both for dynamic as well as for a hepatobiliary phase. The dynamic phases are particularly suitable for assessing the perfusion behavior of focal liver lesions [8, 9]. An additional hepatobiliary phase supports the characterization of focal liver lesions [10].

The OATP-controlled uptake of Gd-EOB-DTPA [11-14] shows normal liver parenchyma specific signal amplification within the parenchyma after 10 minutes to at least 2 hours after intravenous injection [8]. In previous studies, it is shown that the liver function is related to the dynamic contrast agent behavior of MR contrast agents during the vascular phases [15-19] and the uptake of Gd-EOB-DTPA into the liver cells is slower in the case of liver fibrosis [20].

In a retrospective study, we investigated the extent to which the uptake in the hepatobiliary phase is associated with the respective fibrosis or cirrhosis level.For this purpose, 193 patients were examined who underwent MR imaging directly around a histological tissue protection (resection, stamping biopsy) of the liver.

One of the limitations was, among other things, the quality of the investigated liver tissue. 74 patients had to be excluded because of poor histological quality or too little histological material. Furthermore, the liver tissue, which was obtained from resections, showed a better statement about the liver parenchyma and, as such, better matched with MRI data.

References

- Regev, A. et al. Sampling error and intraobserver variation in liver biopsy in patients with chronic HCV infection. The American journal of gastroenterology 97, 2614-2618, doi:10.1111/j.1572-0241.2002.06038.x (2002).
- Germani, G., Hytiroglou, P., Fotiadu, A., Burroughs, A. K. & Dhillon, A. P. Assessment of Fibrosis and Cirrhosis in Liver Biopsies: An Update. Semin Liver Dis 31, 082-090, doi:10.1055/s-0031-1272836 (2011).
- Maharaj, B. et al. Sampling variability and its influence on the diagnostic yield of percutaneous needle biopsy of the liver. Lancet 1, 523-525 (1986).
- Bravo, A. A., Sheth, S. G. & Chopra, S. Liver biopsy. The New England journal of medicine 344, 495-500, doi:10.1056/nejm200102153440706 (2001).
- Cassinotto, C. et al. Liver Fibrosis: Noninvasive Assessment with Acoustic Radiation Force Impulse Elastography--Comparison with FibroScan M and XL Probes and FibroTest in Patients with Chronic Liver Disease. Radiology, doi:10.1148/radiol.13122208 (2013).
- Friedrich-Rust, M. et al. Performance of Acoustic Radiation Force Impulse imaging for the staging of liver fibrosis: a pooled meta-analysis. Journal of viral hepatitis 19, e212-219, doi:10.1111/j.1365-2893.2011.01537.x (2012).
- Bota, S. et al. Intra- and interoperator reproducibility of acoustic radiation force impulse (ARFI) elastography--preliminary results. Ultrasound in medicine & biology 38, 1103-1108, doi:10.1016/j.ultrasmedbio.2012.02.032 (2012).
- Reimer P, Rummeny EJ, Daldrup HE, et al. (1997) Enhancement characteristics of liver metastases, hepatocellular carcinomas, and hemangiomas with Gd-EOB-DTPA: preliminary results with dynamic MR imaging. European radiology 7(2):275-280
- Quillin SP, Atilla S, Brown JJ, Borrello JA, Yu CY, Pilgram TK (1997) Characterization of focal hepatic masses by dynamic contrast-enhanced MR imaging: findings in 311 lesions. Magnetic resonance imaging 15(3):275-285
- Haimerl M, Wachtler M, Zeman F, et al. (2014) Quantitative evaluation of enhancement patterns in focal solid liver lesions with Gd-EOB-DTPA-enhanced MRI. PloS one 9(6):e100315
- van Montfoort, J. E. et al. Hepatic uptake of the magnetic resonance imaging contrast agent gadoxetate by the organic anion transporting polypeptide Oatp1. The Journal of pharmacology and experimental therapeutics 290, 153-157 (1999).
- Weinmann, H. J., Bauer, H., Frenzel, T., Muhler, A. & Ebert, W. Mechanism of hepatic uptake of gadoxetate disodium. Academic radiology 3 Suppl 2, S232-234 (1996).
- Pascolo, L. et al. Molecular mechanisms for the hepatic uptake of magnetic resonance imaging contrast agents. Biochem Biophys Res Commun 257, 746-752, doi:10.1006/bbrc.1999.0454 (1999).
- Nassif, A. et al. Visualization of hepatic uptake transporter function in healthy subjects by using gadoxetic acid-enhanced MR imaging. Radiology 264, 741-750, doi:10.1148/radiol.12112061 (2012).
- Annet L, Materne R, Danse E, Jamart J, Horsmans Y, Van Beers BE (2003) Hepatic flow parameters measured with MR imaging and Doppler US: correlations with degree of cirrhosis and portal hypertension. Radiology 229(2):409-414
- Fischer MA, Donati OF, Reiner CS, Hunziker R, Nanz D, Boss A (2012) Feasibili¬ty of semiquantitative liver perfusion assessment by ferucarbotran bolus injection in double-contrast hepatic MRI. Journal of magnetic resonance imaging : JMRI 36(1):168-176
- Hagiwara M, Rusinek H, Lee VS, et al. (2008) Advanced liver fibrosis: diagnosis with 3D whole-liver perfusion MR imaging--initial experience. Radiology 246(3):926-934
- Tajima T, Takao H, Akai H, et al. (2010) Relationship between liver function and liver signal intensity in hepatobiliary phase of gadolinium ethoxybenzyl diethylenetriamine pentaacetic acid-enhanced magnetic resonance imaging. Journal of computer assisted tomography 34(3):362-366
- Motosugi U, Ichikawa T, Sou H, et al. (2009) Liver parenchymal enhancement of hepa¬tocyte-phase images in Gd-EOB-DTPA-enhanced MR imaging: which biological markers of the liver function affect the enhancement? Journal of magnetic resonance imaging : JMRI 30(5):1042-1046
- Nishie A, Asayama Y, Ishigami K, et al. (2012) MR prediction of liver fibrosis using a liver-specific contrast agent: Superparamagnetic iron oxide versus Gd-EOB-DTPA. Journal of magnetic resonance imaging : JMRI 36(3):664-671

Study goals and objectives

- Presentation of the liver function by means of contrast-enhanced magnetic resonance tomography.
- Detection of the sensitivity and specificity of MRI-related liver parenchymal analysis.

Study Design

Study Characteristics

- Study Type:  Interventional
- Allocation:  Single arm study
- Blinding:  Blinded
- Who is blinded:  assessor
- Control:  Uncontrolled/Single arm
- Purpose:  Diagnostic
- Assignment:  Single (group)
- Phase:  N/A
- Off-label Drug use:  N/A

Interventions/Observational Groups

The degree of liver fibrosis / cirrhosis is determined by means of magnetic resonance tomography with hepatocyte-specific MRI contrast agent and correlated with the histological processing. The different investigators are blinded to the results.

Inclusion Criteria

- Gender:  Both, male and female
- Minimum Age:  18   Years
- Maximum Age:  no maximum age

Additional Inclusion Criteria

Planned elective liver resection. Clinical indication for performing an MRI of the liver with liver-specific contrast medium for preoperative planning.

Exclusion Criteria

Known contrast agent incompatibility. Implants with ferromagnetic metals. Renal insufficiency

Methodology

Radiology/Imaging

All imaging will be performed using a clinical whole-body 3-T system (Magnetom Skyra, Siemens Healthcare) and combination body-spine array coil elements (18-channel body matrix coil and 32-channel spine matrix coil) for signal reception. T1-weighted volume-interpolated breath-hold examination (VIBE) sequences with fat suppression (repetition time (TR), 3.09 ms; echo time (TE), 1.16 ms; flip angle, 9°; parallel imaging factor, 2; slices, 64; reconstructed voxel size, 1.3 × 1.3 × 3.0 mm; measured voxel size, 1.7 × 1.3 × 4.5 mm; acquisition time, 14 s), covering the entire liver, and the sequences will be applied before (non-contrast) and 20 min after contrast injection (HBP). The sequence is acquired during one breath-hold, and no additional system adjustments are performed between sequences.

All patients receive a body weight adapted dose (0.025 mmol/kg body weight) of Gd-EOB-DTPA (Primovist, Eovist; Bayer Schering Pharma AG, Berlin, Germany). The hepatocytic contrast agent Gd-EOB-DTPA is administered via bolus injection with a flow rate of 1 ml/s and flushed with 20 ml NaCl.

The mean SI is calculated using three regions of interest (ROI), manually placed in each liver lobe (with identical sizes and locations in the non-contrast and post-contrast T1 HBP). The RE between non-contrasted (SIpre) and post-Gd-EOB-DTPA (Sipost) is calculated as follows:

Histopathological examination

In the present study, only liver samples of liver resection or transplantation are included to avoid any ambiguities regarding the Ishak score. All samples are fixed in formalin and embedded in paraffin. Sections are cut vertically and mounted on glass slides. After that, the sections are deparaffinised with xylene and ethanol and stained with hematoxylin-eosin (HE) and Elastica van Gieson (EVG) according to standard protocols. EVG staining is used to evaluate LF.

Two pathologists who specialize in liver histopathology review the resection specimens to assess the degrees of fibrosis/cirrhosis. Both readers are blinded to the imaging results and the patient data. The scoring is performed independently. In cases of disagreements, an additional microscopic analysis is done in consensus to find a common final judgment. The fibrosis is classified using the Ishak scoring system.

Safety Considerations

No ionizing radiation is produced within the scope of magnetic resonance tomography so that it is not subject to the X-ray regulation or radiation protection ordinance; A permit issued by the Federal Office for Radiation Protection is not required for carrying out a magnetic resonance tomography.

The contrast agent is used in the clinical routine and is a well-tolerated. It is excreted both by the liver and kidneys, this dual route of excretion is beneficial for patients with impaired liver or renal function since the remaining intact pathway can compensate for the disrupted pathway. Primovist is excreted in the unchanged form via the urine. There is no metabolism or structural alteration of the active substance in the body.

Follow-Up

No further follow-up is planned.

Data Management and Statistical Analysis

We use the nonparametric Mann-Whitney U-test for independent variables for comparisons between groups. All tests are two-sided, and values of p < 0.05 indicated a significant difference. The data of this study are available from the corresponding author upon reasonable request.

Quality Assurance

If patients are not able to complete the full MR imaging protocol or imaging data is corrupted by severe imaging artifacts due to poor breath-holding techniques, these data are being excluded. The limitations of the initial retrospective study are considered in the study design, Especially the quality of the investigated tissue. However, each liver tissue, undergoing histopathological examination, will be subject to a quality reading to ensure quality.

Expected Outcomes of the Study

Correlation between the grade of fibrosis/cirrhosis, classified using the Ishak scoring system, and RE is under investigation and will be compared to an initial retrospective study. A strong coloration between RE and the grade of fibrosis/cirrhosis is affirmed.

Dissemination of Results and Publication Policy

Publication of the project is highly anticipated. If possible, open access is chosen for publication. All participated members will be co-authors in the present study.

Duration of the Project

1 Year

Problems Anticipated

Patients with a change in therapy will be excluded from this study, e.g. no liver resection or atypical liver resection with an insufficient liver sample.

Project Management

N.V. is primarily responsible for this study. He is collecting and interpreting the data. K.U. and M.E. participated in the study design and are collecting the data. M.H., L.B., and C.F. are helping with the data acquisition, and interpretatio. F.Z. is performing the statistical analysis. MH.D. and F.B. revised the project critically for important intellectual content and are responsible for patient acquisition. C.S. and P.W participated in the study design; they are responsible for coordination.

Ethics

- Date of Ethics Committee Application:  2016/07/11
- Date of Ethics Committee Approval:  2016/08/03
- Ethics Approval/Approval of the Ethics Committee:  Approved
- Ethics Committee No.:  16-101-0177,
- Ethics Committee of the University Hospital Regensburg

Approval from the local institutional review board of the University Hospital Regensburg is obtained for this prospective study and performed in accordance with the relevant guidelines and regulations. Written informed consent is obtained from the study participant.
